# The Effects of Intent, Outcome, and Causality on Moral Judgments and Decision Processes

**DOI:** 10.5334/pb.1157

**Published:** 2022-07-04

**Authors:** Aurore Gaboriaud, Flora Gautheron, Jean-Charles Quinton, Annique Smeding

**Affiliations:** 1 Univ. Grenoble Alpes, Univ. Savoie Mont Blanc, LIP/PC2S, F-38000 Grenoble, FR; 2 Univ. Grenoble Alpes, CNRS, Grenoble INP, LJK, F-38000 Grenoble, FR; 3 Univ. Savoie Mont Blanc, Univ. Grenoble Alpes, LIP/PC2S, F-73000 Chambéry, FR

**Keywords:** moral judgment, decision process, intent, outcome, causality, mouse-tracking

## Abstract

Over the past decade, moral judgments and their underlying decision processes have more frequently been considered from a dynamic and multi-factorial perspective rather than a binary approach (e.g., dual-system processes). The agent’s intent and his or her causal role in the outcome–as well as the outcome importance–are key psychological factors that influence moral decisions, especially judgments of punishment. The current research aimed to study the influence of intent, outcome, and causality variations on moral decisions, and to identify their interaction during the decision process by embedding the moral scenarios within an adapted mouse-tracking paradigm. Findings of the preregistered study (final *n* = 80) revealed main effects for intent, outcome, and causality on judgments of punishment, and an interaction between the effects of intent and causality. We furthermore explored the dynamics of these effects during the decision process via the analysis of mouse trajectories in the course of time. It allowed detecting when these factors intervened during the trial time course. The present findings thus both replicate and extend previous research on moral judgment, and evidence that, despite some ongoing challenges, mouse-tracking represents a promising tool to investigate moral decision-making.

The psychology of decision-making applied to the field of morality is a vast and ambiguous topic in the literature, given the many different approaches addressing this issue. It is however an important topic as each of us makes moral decisions in everyday life. During the current Covid-19 crisis for instance, people may wonder whether they need to strictly adhere to recommendations for the greater good even if this impedes individual freedom (e.g., get vaccinated). People may also face moral conflicts when judging other’s uncivil acts, especially if there are extenuating circumstances.

In the current study, we aimed to extend our understanding of the way people judge others’ actions regarding moral situations. When someone commits a harmful action, people judge this agent not only regarding his or her intent, but also regarding the action outcome and the agent’s causal role in this outcome (Cushman, 2008; Martin et al., 2021). We thus analyzed the impact of these three factors on moral judgments and their entanglement in the course of time through a novel use of mouse-tracking paradigms, in an adapted version for the purpose of this study.

## A dynamic and multi-factorial approach to moral decision-making

Decision making constitutes a broad and intensely studied area in moral psychology. Given its multiple approaches, it is sometimes unclear what exactly is studied and under which perspective. As a subtype of general decision-making, moral decision-making is a complex cognitive process of everyday life defined by the ability to make a choice between multiple possibilities to resolve a question in a specific situation (Allain, 2013). Moral judgments (i.e., ‘Is it acceptable to…?’) and moral choices of action (i.e., ‘Would you…’) are conceptually distinct and do not provide the same patterns of results (e.g., FeldmanHall et al., 2012; Gold et al., 2015; Tassy et al., 2013). However, they may yet have a lot in common, as moral judgments also rely to some extent on decision processes (Yu et al., 2019). The frontier between moral judgment and moral decision (as a choice of action) thus remains blurred. We will use the term decision to refer to the generic concept–including both judgment and decision process–whereas judgment will refer to the mere final response.

Historically, moral psychology of decision mainly relied on dual-process models, based on Kahneman’s model of the two speeds of thought (2011). In the face of a moral problem, two cognitive systems compete. One intuitive, fast, emotional, and automatic reflects our inner (i.e., deontological) sense of morality; the other, more controlled, slow, and rational reflects our utilitarian side (i.e., costs vs. benefits ratio) (Greene et al., 2001). This dichotomous approach has been criticized, most notably as we cannot simply assimilate deontological (vs. utilitarian) responses with fast (vs. slow) processes. Both response types can be as intuitive as counterintuitive (Kahane, 2012; Rosas & Aguilar-Pardo, 2020). In addition, deontological and utilitarian concerns are not the only prominent variables influencing moral choices (e.g., Christensen & Gomila, 2012; Leloup et al., 2018). The dual-process model does not well reflect the complexity and richness of cognitive processes underlying moral decisions.

As such, we argue in favor of a dynamic and multi-process model of moral decision (see also Malle, 2021). This multi-factorial perspective supports the conceptualization of moral decision as the result of a combination of numerous dynamic and continuous influences (Spivey & Dale, 2006). We especially aim to analyze moral decision processes depending on the weight taken by various influential factors and by their entanglement over time. Among these, some can be characterized as key factors in moral decision-making.

## Intent and outcome as key factors in moral judgments

The agent’s intent as well as the action outcome are factors that take a preponderant place in morality, from childhood for the outcome and later in the moral development for intent features (Kohlberg, 1958; Martin et al., 2021; Piaget, 1995). Intentional attribution process refers to the inference of the agent’s internal mental states as a combination of beliefs and desires (Cushman, 2008; Cushman, 2015; Cushman & Mele, 2008; Young & Saxe, 2011). Whereas considering the action outcome refers to the perception of the severity of the perpetrated harm (Cushman, 2008; Hirschfeld-Kroen et al., 2021). People indeed judge more severely intentional (vs. non-intentional) acts and harmful (vs. unharmful or neutral) acts (Cushman, 2008; Leloup et al., 2018). Additionally, the agent’s current beliefs regarding the situation seem to matter more in considering his or her intent than the simple desire for causing harm especially for morally wrong actions (Cushman, 2008; Cushman & Mele, 2008).

Regardless of the moral judgment type (i.e., wrongness, permissibility, punishment, or blame), the agent’s intent to cause harm often plays a greater role in the final judgment than considering the outcome of the situation (Cushman, 2008; Kohlberg, 1958; Leloup et al., 2018). Unless the person who judges is under cognitive load, then his or her judgment could be more outcome-based than usual (see Martin et al., 2021). However, even without manipulating cognitive load, outcome importance varies according to the type of moral judgment involved (Cushman, 2008). The outcome would actually be of minimal importance in judgments of wrongness and permissibility (i.e., how much was the perpetrated act wrong or permissible?). It would however explain a much larger part of the variance in judgments of punishment and blame (Cushman, 2008). Leloup et al. (2018) replicated this result with punishment and wrongness judgments using a French-speaking sample responding to typical daily-life moral scenarios.

According to this literature, it would have been expected that manipulating the agent’s intent and the mere presence (vs. absence) of a harmful outcome would be sufficient to observe an interaction effect between intent and outcome. That is, the strong effect of intent on moral judgments would be even stronger when the action results in a harmful outcome (vs. neutral outcome). However, this interaction effect proved to be significant only when another factor–often confounded within the outcome factor–was explicitly manipulated: the agent’s causal role in the outcome (Cushman, 2008, study 3). Often indeed, when the outcome is manipulated (e.g., neutral vs. harmful outcome), there is no explicit information regarding who or what did cause the actual outcome. Knowing that the outcome was caused by the agent or by another source–even sometimes by the victim him or herself–seems to represent another key factor in the decision, and will be manipulated in a novel way in the present research.

## Causality as another key factor and moderator in moral judgments

The causal attribution process corresponds to the inference of the agent’s causal role in the outcome of the action, grounded on Heider’s theory of attribution (1958, see also Cushman, 2008; Cushman & Young, 2011). The outcome is either directly caused by the agent’s action or caused by another source. As evidenced by Cushman (2008), the agent’s causality takes its strongest influence on moral decisions when a judgment of punishment or blame is at stake (compared to judgments of wrongness or permissibility). In these two types of moral judgments (i.e., punishment and blame), the causality factor did impact the strength of the intent effect on the final decision (Cushman, 2008, study 3). This interaction effect was however weak, which questions its generalizability, especially as Cushman (2008) did not use mixed models to test it. As morality is a domain where there can be a lot of variability–both at the participant and stimulus levels–it seems highly relevant to model the random effects related to the participants and stimuli, which allows for generalization.

Based on these results, Cushman (2008) developed a model with two distinct processes of moral judgments. One involved in wrongness and permissibility judgments with inferences mainly about the agent’s intent; the other involved in blame and punishment judgments with inferences about both the agent’s intent and causality in the outcome (Cushman, 2008). The main effects of intent, outcome, and causality on moral judgments are therefore rather well-established, while their interactive effects are less clear.

Furthermore, as moral decisions can be conceived as a continuous and dynamic process (Koop, 2013; Spivey & Dale, 2006), we suggest that the influences of intent, outcome, and causality would result in distinguishable dynamics in decision over the course of time. This was the case for personal vs. impersonal dilemmas, as evidenced by Koop in 2013 with a mouse-tracking paradigm. Mouse-tracking is a relevant technique to study the underlying decision process in its entirety (i.e., not based only on the final response) and to give access to continuous real-time cognition through hand movements (Freeman & Ambady, 2010; Freeman et al., 2011; Spivey & Dale, 2006). Although mouse-tracking paradigms have almost never been used for moral scenarios (except by Koop in 2013)–not the least because such scenarios are often lengthy and thus ill-adapted to mouse-tracking–some features of previous studies (e.g., Leloup et al., 2018) suggest that a mouse-tracking paradigm can be used to study moral scenarios.

## Overview of the current study

In the present research, we aimed to conceptually replicate and extend previous findings regarding the influence of intentional and causal factors on moral judgments of punishment (Cushman, 2008; Leloup et al., 2018). By adapting Leloup et al.’s procedure (2018) to suit mouse-tracking, we first tested the replicability of the main effects of intent, outcome, and causality on judgments of punishment with this new paradigm. As these main effects–and that of intent in particular–have quite systematically been found in previous research and if the present new paradigm does not substantially modify moral decisions, we should at least replicate these main effects on participants’ judgments. Second, to clarify the robustness and meaning of the intent-by-causality interaction, we created three conditions for the outcome variable (i.e., neutral outcome, harmful outcome either caused or not caused by the agent) resulting from the adaptation of Samson and Leloup’s (2018) French materials and from the improvement of Cushman’s (2008) initial design. This feature was particularly important, as it allowed testing whether the agent’s causality in the outcome or the outcome itself matters more in moral judgments of punishment, hence possibly extending Cushman’s model (2008). Further extending and refining previous research (Cushman, 2008), mixed models were systematically used to better take into account the variability inherent to morality, both at the participant and stimulus levels. Finally, we relied in an exploratory and innovative vein on the mouse-tracking data to examine when exactly during the decision process the effects of intent, outcome, causality, and their interactions appeared–as mouse trajectories reflect the course of mind (see Freeman & Ambady, 2010; Spivey & Dale, 2006).

For this purpose, we simplified Leloup et al.’s (2018) procedure by removing the factor of order of information presentation and by only focusing on judgments of punishment. These were indeed those–with blame judgments–that triggered to a greater extent the outcome and causality components, in comparison to judgments of permissibility and wrongness (Cushman, 2008). As Leloup et al.’s study (2018) only comprised punishment and not blame judgments, we only kept the former for direct comparison purposes, given the other changes implemented in the paradigm due to classic mouse-tracking requirements (which are not met in the case of moral scenarios and decisions).

We expected judgments of punishment to be more severe when the agent’s action was intentional (vs. non intentional), had a harmful outcome (vs. neutral), and when the outcome was caused by the agent (vs. not caused by him or her). Furthermore, we predicted that the effect of intent on judgments of punishment would depend on knowledge of the actual outcome on the one hand and of its causal origin on the other hand (i.e., interaction effects).

We thus conducted an experiment where participants responded via the computer mouse to various moral scenarios manipulating the agent’s intent, the outcome, and the agent’s causality in a counterbalanced design, while recording the mouse coordinates on the screen over time. Participants provided informed consent before participation and the project was approved by the local ethics committee. We reported all measures, manipulations, and exclusions. This study was pre-registered and all additional materials (i.e., preregistration form, power analysis, scenarios database, data files, and analyses script) are available on the project OSF page (see the section ‘Additional Files’).

## Method

### Power analysis

To determine the required sample size for this study, we conducted a power analysis through PANGEA (Westfall, 2016). For intent and outcome variables, we estimated power based on the effect sizes obtained by Leloup et al. (2018) and recomputed through linear mixed models based on their data (see ‘Additional Files’ section for more). We found that a power of 90% would be reached with a sample of *n* = 40. For the causality variable, based on Cushman’s results (2008), a power of 80% would be reached with *n* = 40 and of 98% with *n* = 80 participants. The intent-by-causality interaction accounted for 5% of the variability in judgments of punishment (Cushman, 2008), corresponding approximatively to *R*^2^ = 0.05 equivalent to *d* = 0.46. According to Westfall et al. (2014), a power of 80% for such an effect size and with a counter-balanced design is reached with *n* = 32.

We chose to fix a reference sample size of *n* = 80. As effect sizes were uncertain given the implemented changes, and following Lakens’ recommendations (2014), we conducted a sequential analysis. Based on an a priori maximum sample size of 120 participants, we conducted intermediate analyses at 40 and 80 participants. If all main effects of interest were observed at *n* = 40 (with an adjusted alpha threshold at 0.017) or at *n* = 80 (adjusted alpha at 0.022), data collection would be stopped. As *p*-values for the main effects were estimated far below adjusted alpha threshold with 80 participants, we stopped data collection and did not apply additional corrections.

### Participants

Eighty Psychology undergraduates (*n* = 69 females) participated in exchange for course credit. Data were collected within two sessions after preregistration (i.e., March and October 2020) due to the Covid-19 crisis and subsequent lockdown in France. We therefore conducted the first intermediate analysis at the end of the first session (i.e., with 37 participants) and the second analysis on the total sample of 80 participants (four additional participants were excluded beforehand because they had already participated to the experiment during the first session).

### Materials

The original materials from Leloup et al.’s article (2018), composed of 64 moral hypothetical scenarios (see Samson & Leloup, 2018, CC-BY 4.0), were adapted for the purpose of this study. The four original versions of each scenario manipulating the agent’s intent (intentional vs. non-intentional) and the presence of a harmful outcome (harmful vs. neutral) were taken unaltered from Leloup et al.’s study (2018). To test the effect of causality, we created another condition within the outcome ‘harmful’ modality: the agent’s causal role in this harmful outcome (either caused by the agent or not caused by him or her). This resulted in six (2 × 3) variants for each scenario (example in Table 1). Note that some scenarios have been slightly rephrased to better suit a French sample–as participants in Leloup et al.’s (2018) study were Belgian (see the entire materials in the ‘Additional Files’ section).

**Table 1:** An example of the six versions of the same scenario adapted from Leloup et al. (2018).


VERSION OF THE SCENARIO	NOT INTENTIONAL & NEUTRAL OUTCOME (‘NEUTRAL’)	NOT INTENTIONAL & HARMFUL OUTCOME (‘ACCIDENTAL HARM’)

Step 0. Context	Cindy and Valerie decide to have a drink after work. Valerie has to drive her car to go back home. Valerie asks Cindy to order her a non-alcoholic cocktail while she goes to the bathroom. Cindy orders a homemade cocktail for Valerie.	Cindy and Valerie decide to have a drink after work. Valerie has to drive her car to go back home. Valerie asks Cindy to order her a non-alcoholic cocktail while she goes to the bathroom. Cindy orders a homemade cocktail for Valerie.

Step 1. Intent	Cindy thought the homemade cocktail was not alcoholic.	Cindy thought the homemade cocktail was not alcoholic.

Step 2. Outcome	Valerie is in condition to drive and does not have any accident on the way back home.	Valerie is not in condition to drive and has an accident on the way back home.

**VERSION OF THE SCENARIO**	**INTENTIONAL & NEUTRAL OUTCOME (‘ATTEMPTED HARM’)**	**INTENTIONAL & HARMFUL OUTCOME (‘INTENTIONAL HARM’)**

Step 0. Context	Cindy and Valerie decide to have a drink after work. Valerie has to drive her car to go back home. Valerie asks Cindy to order her a non-alcoholic cocktail while she goes to the bathroom. Cindy orders a homemade cocktail for Valerie.	Cindy and Valerie decide to have a drink after work. Valerie has to drive her car to go back home. Valerie asks Cindy to order her a non-alcoholic cocktail while she goes to the bathroom. Cindy orders a homemade cocktail for Valerie.

Step 1. Intent	Cindy thought the homemade cocktail was alcoholic.	Cindy thought the homemade cocktail was alcoholic.

Step 2. Outcome	Valerie is in condition to drive and does not have any accident on the way back home.	Valerie is not in condition to drive and has an accident on the way back home.

**VERSION OF THE SCENARIO**	**INTENTIONAL & NOT CAUSED HARMFUL OUTCOME (‘NON-CAUSED BUT INTENTIONAL HARM’)**	**NOT INTENTIONAL & NOT CAUSED HARMFUL OUTCOME (‘NON-CAUSED & NON-INTENTIONAL HARM’)**

Step 0. Context	Cindy and Valerie decide to have a drink after work. Valerie has to drive her car to go back home. Valerie asks Cindy to order her a non-alcoholic cocktail while she goes to the bathroom. Cindy orders a homemade cocktail for Valerie.	Cindy and Valerie decide to have a drink after work. Valerie has to drive her car to go back home. Valerie asks Cindy to order her a non-alcoholic cocktail while she goes to the bathroom. Cindy orders a homemade cocktail for Valerie.

Step 1. Intent	Cindy thought the homemade cocktail was alcoholic.	Cindy thought the homemade cocktail was not alcoholic.

Step 2. Outcome	Valerie is in condition to drive but she hits an animal on the road and has an accident.	Valerie is in condition to drive but she hits an animal on the road and has an accident.


*Note*: The first four versions of the scenario (i.e., *Neutral; Accidental Harm; Attempted Harm; Intentional Harm*) were kept unaltered from the original material (Samson & Leloup, 2018). The last two were created within the same logic but adapted according to the model proposed by Cushman (2008, Experiment 3).

### Procedure and design

Upon arrival, participants signed a consent form and were seated in individual cubicles equipped with a computer. The procedure was mainly based on Leloup et al.’s experiment (2018), but was embedded into an adapted mouse-tracking paradigm (see Freeman & Ambady, 2010 for a classic mouse-tracking paradigm). All responses were given with the computer mouse, calibrated so that the speed of the pointer was limited (with pointer acceleration switched off).

**Figure 1:**
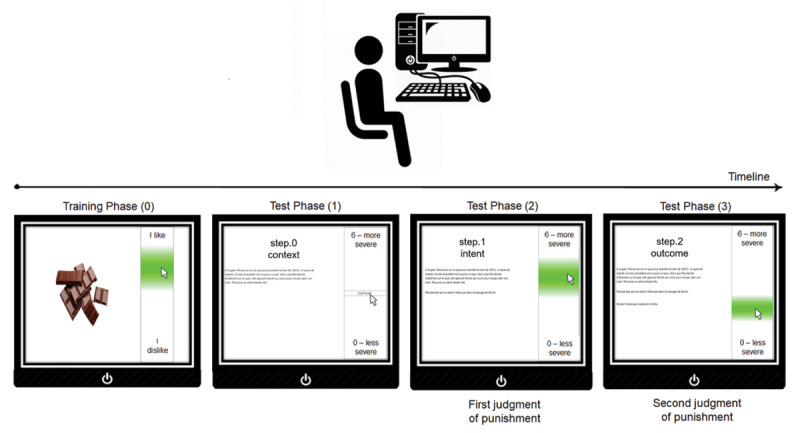
Schematic procedure of the current mouse-tracking paradigm. *Note*: The mouse pointer was not displayed during the trials but was included here in the figure for illustrative purposes.

After a brief training session to get familiar with the interface (i.e., 6 non-moral and 4 moral trials–taken from the original set of scenarios), participants went through the 60 remaining moral scenarios. Each trial (i.e., one scenario) was completed in three successive steps: first, participants only read the context of the scenario without time pressure (step 0); second, they received additional information about the agent’s intent and had to provide a first judgment of punishment targeting the agent (step 1); third, participants received final information about the action outcome and had to give a second judgment of punishment accordingly (step 2) (see Figure 1). For steps 1 and 2, participants allocated a certain number of penalty tokens ranging from 0 (*less severe*) to 6 (*more severe*) to the agent, figuring the severity of their judgments of punishment. Within a same scenario, the number of tokens was reset at the beginning of each step, so that participants were not forced to distribute a fixed set of tokens across the two steps or across trials. Moreover, the pointer position was reset at the beginning of each step (back to the middle of the vertical scale). The two steps could then be considered as independent from a paradigmatic viewpoint. However, from the participant perspective, step 2 was inherently linked to step 1 as the same situation was displayed during both steps, hence a possible influence of step 1 responses on step 2 responses may have emerged. Participants were also required to continuously move the computer mouse (see Hehman et al., 2015). Trials were considered as missed if the participants did not make any move with the mouse at the trial onset. Every 12 trials, a self-paced break was introduced and a short reminder of the instructions was provided.

The study followed a within-participants and within-stimuli counter-balanced design. Participants randomly went through each of the 60 scenarios in only one of the six possible versions (i.e., 10 scenarios in each condition, randomly selected to avoid any block effect). Each participant judged a same scenario only once and processed each condition about 10 times. Each scenario was randomly presented in each of the six conditions, but each participant only saw each scenario in a single condition (i.e., counter-balanced across participants), crossing the intent (2: intentional vs. non-intentional) and outcome (3: neutral vs. not caused by agent vs. caused by agent) variables. The main dependent variable was the judgment of punishment figured by the attributed number of tokens for each scenario, either in step 1 (i.e., when only intent was known) or in step 2 (i.e., when both intent and outcome were known). Mouse movements–Y-coordinates in the course of time–were recorded for the exploratory analyses. These Y-coordinates were rescaled from 0 to 6, in order to match the judgment response scale of the final answers.

## Results

### Analytical strategy

All analyses (i.e., confirmatory and exploratory) were conducted with *R* programming language (*RStudio*, version 1.3.1093). *lme4* package (Bates et al., 2015) and some other packages (e.g., *lmerTest, emmeans, car*) were used. Linear mixed models (LMM) were used to better account for large inter-participant and inter-stimuli variabilities, allowing at the same time the generalization of the results to both other participants and stimuli (i.e., other moral scenarios) (Judd et al., 2012). We considered two fixed factors (i.e., intent and outcome) and two random factors (i.e., participant ‘P’ and stimulus ‘S’). We recoded intent by the following contrast: [int] non-intentional (–0.5) vs. intentional (0.5); and the outcome variable by Helmert contrasts: We named the first one the ‘outcome’ contrast–[out.O] not caused (1/3) and caused (1/3) vs. neutral (–2/3)–to analyze the effect of the mere presence of a harmful outcome. The second one is the ‘causality’ contrast–[out.C] not caused (–0.5) vs. caused (0.5) vs. neutral (0)–to analyze the effect of the agent’s causality on moral judgments.

We then applied a procedure for reducing the random structure of the model to prevent incorrect estimates of statistics–without altering the specification of fixed effects–following the recommendations of Bates et al. (2018). With little variations in the fixed effect estimates relatively to the maximal model, we obtained the following parsimonious model whose random structure was supported by the data (in *R* equation format): Judgment ~ int*(out.O+out.C) + (int*out.C+out.O | P) + (int*out.C | S). Fixed effects were thus estimated for intent, for both contrasts of outcome, as well as their interactions, while only some of these effects were kept as random effects, in addition to all correlations between random estimates for both participants [P] and stimuli [S].

We calculated the *p*-values associated with the different effects of interest using a Satterthwaite approximation for degrees of freedom estimation. Alpha threshold was set at 0.05 to determine significance of the effects. Effect sizes are reported as Cohen’s *d*, adapted to linear mixed models (Judd et al., 2017). We excluded from the analysis all missed trials and trials with a response time above 10s. Moreover, two outliers on the participant random effects were detected. They were removed for increased validity of the estimates, while controlling for type I error rate, leaving a final sample of 78 participants and 60 scenarios (see the analysis script for more, available from the section ‘Additional Files’). Table 2 provides a summary of the descriptive results for judgments of punishment in each condition in steps 1 and 2.

**Table 2:** Descriptive statistics for judgments of punishment in steps 1 and 2 across conditions.


CONDITION		JUDGMENTS IN STEP 1	JUDGMENTS IN STEP 2
	
INTENT	OUTCOME	*M (SE)*	*M (SE)*

Intentional	Caused	4.07 (0.12)	4.56 (0.12)

	Not Caused	3.96 (0.12)	3.50 (0.12)

	Neutral	4.01 (0.12)	3.19 (0.13)

Non-intentional	Caused	1.96 (0.12)	2.77 (0.13)

	Not Caused	1.86 (0.11)	1.97 (0.12)

	Neutral	1.89 (0.11)	1.47 (0.11)


*Note*: Judgments in step 1 correspond to judgments of punishment when only intent was available. Judgments in step 2, when both intent and outcome were available. Higher scores (on a 0–6 range) reflect more severe judgments of punishment.

### Confirmatory analyses

We expected judgments of punishment to be more severe (a) when the action was intentional (vs. non-intentional) in steps 1 and 2; (b) when there was a harmful outcome (vs. neutral outcome) in step 2; and (c) when the outcome was caused by the agent (vs. not caused by him or her) in step 2. We additionally predicted that the effect of intent on judgments of punishment in step 2 would be stronger when there was a harmful outcome (intent-by-outcome interaction) and when the outcome was caused by the agent (intent-by-causality interaction). Although confirmatory, previous research did not systematically find the intent-by-outcome interaction (Cushman, 2008; Leloup et al., 2018).

**Figure 2:**
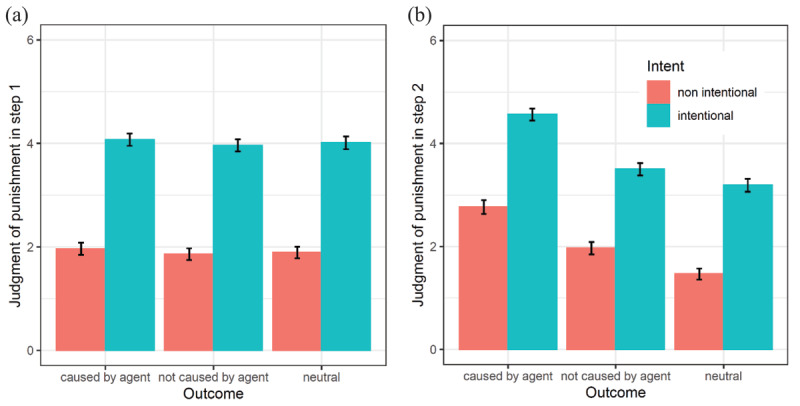
Judgments of punishment in step 1 **(a)** and in step 2 **(b)** depending on intent and outcome. *Note*: Judgments in step 1 correspond to judgments of punishment when intent only was available; judgments in step 2, when both intent and outcome were available. Error bars represent *SE*.

As predicted, results from the mixed model analysis indicated in step 1 (i.e., with only intent information being available) a significant effect of intent, *b* = 2.11, *SE* = 0.17, *t*(88.99) = 12.12, *p* < .001, 95% CI [1.76, 2.46], *d* = 1.40, with people judging more severely the agent when he or she intentionally committed the wrong action (*M* = 4.02, *SE* = 0.12) rather than non-intentionally did so (*M* = 1.91, *SE* = 0.11) (see Figure 2, (a)). We also found an unexpected effect (but of lower magnitude) of the agent’s causality in the outcome, *b* = 0.11, *SE* = 0.05, *t*(55.78) = 2.29, *p* = .03, 95% CI [0.01, 0.20], *d* = 0.07, with judgments of punishment being slightly more severe when the outcome was caused by the agent (*M* = 3.02, *SE* = 0.08) rather than caused by another source (*M* = 2.91, *SE* = 0.07). This effect was quite surprising given that the participant was still unaware of the action outcome in step 1. It was however a posteriori explained as the result of contingencies through the sequence of scenarios. Indeed, despite our randomization efforts across participants and trials, there was a weak non-significant dependency between causality on subsequent trials (*OR* = 0.87, *SE* = 0.09, *z* = –1.34, *p* = .18). This made it possible for the participants to pick up and exploit such contingency between step 2 of one trial and step 1 of the next trial (see the analytic code for more, section ‘Additional Files’). No other main or interaction effect was found at this step.

In step 2, when information of both intent and outcome was available, a significant effect for intent was again found, *b* = 1.68, *SE* = 0.16, *t*(83.25) = 10.60, *p* < .001, 95% CI [1.37, 2.00], *d* = 1.05, with judgments of punishment being more severe when the action was intentional (*M* = 3.75, *SE* = 0.10) rather than non-intentional (*M* = 2.07, *SE* = 0.10) (see Figure 2, (b)). Moreover, as expected in this step, a significant effect was observed for the presence of a harmful outcome–be it caused or not caused by the agent–, *b* = 0.87, *SE* = 0.08, *t*(76.23) = 10.76, *p* < .001, 95% CI [0.71, 1.03], *d* = 0.54, with moral judgments being more severe when there was a harmful outcome (*M* = 3.20, *SE* = 0.07) compared with a neutral outcome (*M* = 2.33, *SE* = 0.08). We additionally found a significant effect for causality, *b* = 0.93, *SE* = 0.10, *t*(79.25) = 9.56, *p* < .001, 95% CI [0.74, 1.13], *d* = 0.58. The agent was indeed punished more severely when his or her action directly caused the outcome (*M* = 3.67, *SE* = 0.09) compared to when the outcome was caused by another source (*M* = 2.73, *SE* = 0.08) (see Figure 2, (b)).

Contrary to expectations, there was no significant intent-by-outcome interaction, *b* = –0.06, *SE* = 0.08, *t*(3782.78) = –0.71, *p* = .48, 95% CI [–0.22, 0.10], *d* = –0.02. However, aligned with expectations, a significant interaction between intent and causality was observed, *b* = 0.26, *SE* = 0.11, *t*(65.65) = 2.34, *p* = .02, 95% CI [0.04, 0.49], *d* = 0.08. The effect of intent on judgments of punishment did depend on the agent’s causality in the outcome. The intent effect became even stronger (+0.26) as the outcome was caused (vs. not caused) by the agent (see Figure 2, (b)). Paired comparisons showed that when the action was caused by the agent, people judged more severely intentional acts (*M* = 4.56, *SE* = 0.12) than non-intentional ones (*M* = 2.77, *SE* = 0.13), *b* = 1.80, *SE* = 0.16, *t*(89.90) = 10.99, *p* < .001, 95% CI [1.47, 2.12]. Similarly, when the outcome was not caused by the agent, participants punished significantly more severely the agent when he or she intentionally acted (*M* = 3.50, *SE* = 0.12) compared to scenarios when he or she non-intentionally acted (*M* = 1.97, *SE* = 0.12), *b* = 1.53, *SE* = 0.18, *t*(82.71) = 8.61, *p* < .001, 95% CI [1.18, 1.89].

### Exploratory analyses

**Figure 3:**
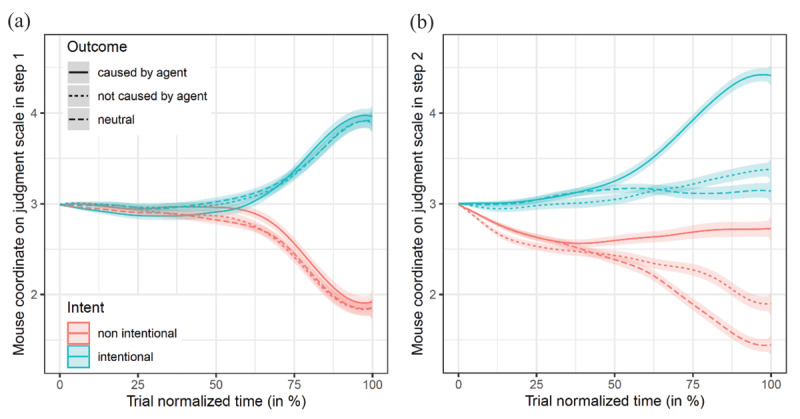
Effects of intent, outcome, causality, and their interactions depending on the time course in step 1 **(a)** and in step 2 **(b)**. *Note*: Y-coordinates were rescaled between 0 (*less severe*) and 6 (*more severe*), so that results at the end of trajectories match the judgment scale of Figure 2. Error bars represent *SE*.

For exploratory analyses on the mouse-tracking trajectories, we rescaled Y-coordinates between 0 and 6, so that they directly reflect the severity of judgments (see Figure 3). Although coordinates alone are not sufficient to infer the aimed judgment during the decision process, we expected the following patterns with (a) a strictly positive effect of intent during the time course of step 1 and from an early stage of step 2–as step 1 should serve as a prime for the next step; (b) at the end of step 2, lower values for intent effect compared to the beginning–as the influence of intent should be weighted down by the outcome and causality information; (c) strictly positive effects of outcome and causality in step 2; (d) at the end of step 2, a larger effect of intent compared to both outcome and causality effects (respecting the differences in raw and standardized effect sizes observed on final judgments).

To explore those patterns, we selected 93% of the mouse trajectories, excluding those which did not meet standard mouse-tracking assumptions or led to violations of statistical assumptions (see analytic code for more, section ‘Additional Files’). Mixed models were applied on rescaled Y-coordinates for each step, using the same parsimonious model as for the analysis on final responses. To control for multiple testing through timesteps, analyses were performed on splines coefficients, each focusing on a portion of time-normalized trajectories.^1^ Time therefore runs between 0% when clicking on the START button to 100% when clicking on the final response. Apart from small deviations due to the integration of information through time and to the type of functional statistics used for analyzing trajectories, results at 100% of trajectory time should match those reported in confirmatory analyses on final judgments.

These analyses revealed that the effect of intent became significant at 55% of step 1, *b* = 0.11, *SE* = 0.05, *t*(55.02) = 2.05, *p* = .04, 95% CI [0.003, 0.21], *d* = 0.07. It became stronger during the time course, until reaching its maximal effect size at the end of step 1, *b* = 2.05, *SE* = 0.17, *t*(88.88) = 11.99, *p* < .001, 95% CI [1.71, 2.39], *d* = 1.39 (see Figure 3, (a)). In step 2 however, the intent effect was significant from the beginning of the trial (i.e., earlier than 5% of the whole trajectory duration), *b* = 0.009, *SE* = 0.003, *t*(63.46) = 2.59, *p* = .012, 95% CI [0.002, 0.02], *d* = 0.10; and became stronger until the end, *b* = 1.62, *SE* = 0.16, *t*(82.13) = 10.30, *p* < .001, 95% CI [1.31, 1.93], *d* = 1.03 (see Figure 3, (b)).

The presence of a harmful outcome had no significant effect in step 1 (as expected), while it became significant at 60% of the time course in step 2, *b* = 0.16, *SE* = 0.05, *t*(85.06) = 3.22, *p* = .002, 95% CI [0.06, 0.26], *d* = 0.12; and became stronger until the end, *b* = 0.81, *SE* = 0.08, *t*(74.97) = 10.44, *p* < .001, 95% CI [0.66, 0.97], *d* = 0.51 (see Figure 3, (b)). The causality effect was found as significant in step 1 for only a brief part of the trajectory, between 75% and 80%, *b* < 0.12, *SE* > 0.05, *t* < 2.63, *p* > .010, *d* < 0.08 (see Figure 3, (a)); thus confirming the weakness of this effect found in confirmatory analysis for final judgments in step 1. In step 2 however, the causality effect became significant at 20% of normalized time, earlier than the outcome effect, *b* = 0.08, *SE* = 0.03, *t*(67.36) = 2.53, *p* = .01, 95% CI [0.02, 0.14], *d* = 0.12, and increased until the end of the trial, *b* = 0.93, *SE* = 0.10, *t*(80.00) = 9.28, *p* < .001, 95% CI [0.73, 1.13], *d* = 0.59. The intent-by-causality interaction became significant later in the decision process, at 70%, *b* = 0.26, *SE* = 0.11, *t*(65.46) = 2.42, *p* = .02, 95% CI [0.04, 0.47], *d* = 0.09. Its effect increased until 80% of the whole trajectory duration with *b* = 0.36, *SE* = 0.11, *t*(55.18) = 3.17, *p* = .003, 95% CI [0.13, 0.60], *d* = 0.12; and was then attenuated until the end of the trial, *b* = 0.21, *SE* = 0.11, *t*(55.99) = 1.89, *p* = .06, 95% CI [–0.01, 0.43], *d* = 0.07 (see Figure 3, (b)).

## Discussion

The present study was designed to conceptually replicate and extend previous findings about intent, outcome, and causality influences on moral judgments of punishment (Cushman, 2008; Leloup et al., 2018) through a novel use of mouse-tracking paradigms, which has so far only rarely been used to explore moral decision-making. Findings support the main hypotheses, namely that people judge the agent more severely when the action is intentional (vs. non-intentional), when it induces a harmful outcome (vs. a neutral outcome), and when this outcome is caused by the agent (vs. not caused by him or her). These main effects are consistent with previous findings (e.g., Cushman, 2008; Leloup et al., 2018) and allow to reaffirm those variables as key factors for moral decision-making.

Regarding interaction effects, we did not find a significant interaction between intent and outcome factors in step 2, but the expected interaction between intent and causality factors. Together, these findings are consistent with previous results (e.g., Cushman, 2008; Leloup et al., 2018) that did not systematically find the intent-by-outcome interaction but showed a significant intent-by-causality effect (Cushman, 2008). The present results support Cushman’s hypothesis (2008) that the causal attributional process influences the intentional one, at least for judgments of punishment, whereas the mere presence of a harmful outcome does not. Extending Cushman’s model of moral judgment (2008), the causality of the outcome seems to matter more for the final decision of punishment than the outcome itself. This finding should thus be integrated to Cushman’s two process model of moral judgment (2008) with a clear distinction between outcome and causality concerns. Future research should test whether the same interaction effect is to be found with judgments of blame, which are hypothesized to trigger the same reasoning process than judgments of punishment in Cushman’s model (2008).

Beyond the contribution related to findings on final judgments, the exploratory analyses on mouse trajectories evidenced that intent, outcome, and causality did not influence the decision process at the same time or with the same magnitude. As the intent information appeared first in the current paradigm, there was first a predominant importance of this factor whereas factors of outcome and causality weighed only later in the trial (from the middle of step 2). In addition, the three main effects took more and more importance in the decision during the time course (stronger effects as the time goes by). The intent effect appeared very early in step 2, as if its high importance in the end of step 1 served as a prime in the next step. Additionally, intent weighed to a lesser extent at the end of step 2 compared to the end of step 1, a finding that is consistent with the competition created by the outcome and causality factors, which reduced its influence. Finally, at the end of step 2, intent weighed to a greater extent compared to causality and outcome factors. It supports the idea, with a different measure than previously investigated (i.e., an adapted mouse-tracking paradigm), that intent explains a larger part of variance than outcome or causality in judgments of punishment (Cushman, 2008). We should, however, note that the comparison between step 1 and step 2 judgments and processes is quite tricky as the amount of information–and thus the difficulty of the decision–were not identical throughout the two steps, and what we measured was thus necessarily different between both steps. Step 1 judgments with only intent being manipulated could sometimes elicit some uncertainty as the outcome part was not already displayed. But it does not seem to have impeded participants’ understanding of the situations, as they had to judge sequentially multiple scenarios with the same structure and could expect as soon as step 1 was displayed that a possible consequence was going to happen in step 2.

Furthermore, having performed two-random-variables mixed model to analyze these effects allows their generalization to both other participants and other moral stimuli, extending this way the scope of previous work. Nonetheless, we should admit that the currently targeted population (i.e., undergraduated students) was not representative of the general population, thus nevertheless somewhat restricting the generalization of the present results. We could however easily reproduce this experiment with a general population, as the current mouse-tracking paradigm is very intuitive, user-friendly, not expensive, and easy to implement on a large scale. These are some advantages that the mouse-tracking tool offers to collect functional data in comparison to other techniques (e.g., eye-tracking) (Rivollier et al., 2021). Moreover, we should recognize that the material we used, adapted from Leloup et al.’s study (2018), had certain limitations. Some scenarios were not always sufficiently explicit especially regarding the outcome information (both in the original material and in our adaptation). This could have partially attenuated the effects of outcome and causality, but this seems not to have heavily impacted the decision process as we still observed strong effects for those variables (for which we did not expect an influence as strong as that of intent).

In this vein, beyond getting a first grasp of the temporal influence of intent, outcome, and causality factors during moral decision-making, the present research provides evidence for the applicability of a mouse-tracking paradigm to the moral domain. Having found similar results as Cushman (2008, Study 3) and Leloup et al. (2018, Study 1) for intent, outcome, and causality main and interaction effects supports the idea that the current mouse-tracking paradigm did not significantly alter these expected effects. Embedding lengthy moral scenarios into a mouse-tracking paradigm is not trivial though, as reading entire sentences is ill-suited for typical mouse-tracking studies (e.g., Freeman & Ambady, 2010). This may explain why, despite being a promising tool to investigate moral decisions, mouse-tracking has been so far very rarely used in this domain (see Koop, 2013 for an exception). The present adapted mouse-tracking paradigm seems however suitable for this endeavor, as hypothesized effects were replicated and further specified in terms of their temporality during the decision process. Even if the current exploratory results on mouse-tracking data would need to be replicated in future research in a confirmatory vein, we argue in favor of harnessing the potential of this tool to have a more in-depth understanding of the complexity of human moral decision-making.

## Conclusion

Using a mouse-tracking adapted paradigm and relying on an improved experimental design, findings of the present research both align with and extend previous studies on the preponderant influence of intent in our daily moral decisions. When we are dealing with a morally ambiguous situation in life, we first want to know what was the real intent of the agent that led to this (bad) action. However, intent is not alone, as we demonstrated the progressive integration of outcome information into the decision process, though its effect remains more tenous than intent in punishment decisions. Present findings further indicate that we give even more importance to the agent’s intent when his or her behavior is the direct cause for the observed outcome, more so than the presence of a harmful outcome per se.

## Data Accessibility Statement

The complete data and analytic code used for the analyses of the current study can be found in ‘Data Analyses’ & ‘Raw Data’ sections respectively, available from the OSF link below, in Additional Files.

## Additional File

The additional file for this article can be found in the project OSF page: https://osf.io/3uyps/?view_only=525e16c347b544b0bbde6aae8d3947f7.

## Footnotes

Splines are similar to timebins, but respect the functional nature of the mouse-tracking data (i.e., continuity of hand movements) and estimate effects as a function of time (i.e., mean effect trajectories and associated *SE*, as represented on Figure 3).

## Author Information

This article is partially based on the first author’s unpublished Master’s dissertation.
